# Supplementary material for: Development of a Novel AAV Gene Therapy Cassette with Improved Safety Features and Efficacy in a Mouse Model of Rett Syndrome
Source: Mol Ther Methods Clin Dev. 2017 Apr 22;5:180–90. doi: 10.1016/j.omtm.2017.04.007 (PMC5423329; doi:10.1016/j.omtm.2017.04.007)
Supplement: Document S1. Figures S1–S7 [file mmc1.pdf]

OMTM, Volume *5*

Supplemental Information

Development of a Novel AAV Gene Therapy Cassette with Improved Safety Features and Efficacy in a Mouse Model of Rett Syndrome

Kamal K.E. Gadalla, Thishnapha Vudhironarit, Ralph D. Hector, Sarah Sinnett, Noha G. Bahey, Mark E.S. Bailey, Steven J. Gray, and Stuart R. Cobb

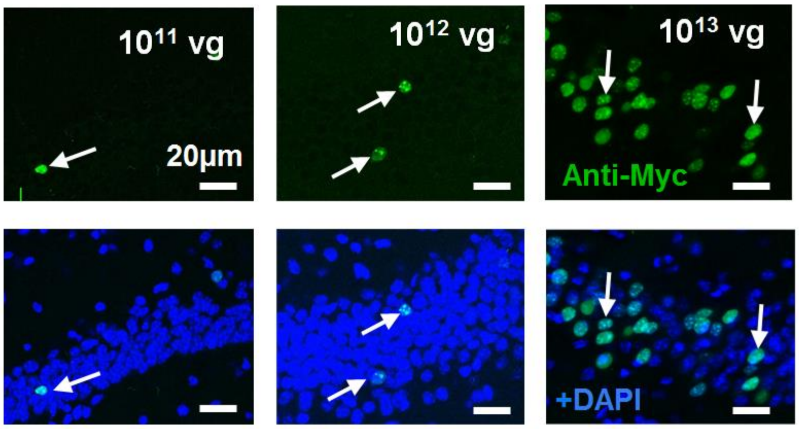

Figure S1. Expression of vector-derived MeCP2 in the brain after intravenous injection of the 1st generation vector.

Representative confocal micrographs showing transgene expression in the hippocampal CA1 region in *Mecp2$^{-/y}$* mice treated intravenously with 1x10^{11},1x10^{12} and 1x10^{13} vg/mouse of the 1st generation vector (as revealed by anti-Myc tag immunolabelling). Arrows denote transduced cells and the lower panel shows co-localisation with DAPI. Scale bar = 20 µm

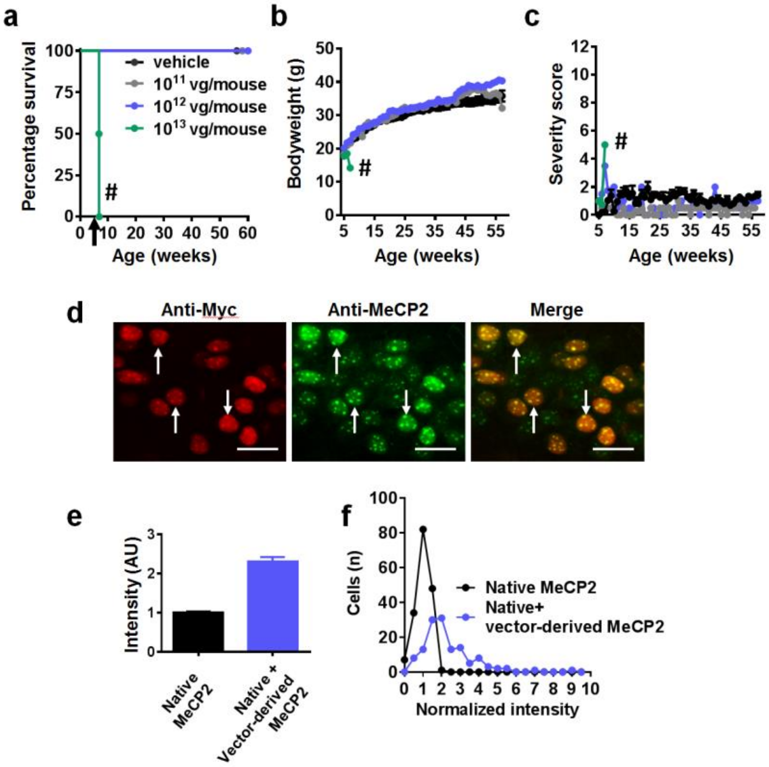

Figure S2. Systemic delivery of the 1st generation vector to wild-type mice is tolerated at low doses but toxic at high doses.

(a) Survival plot showing the early toxicity observed after IV injection of a $1x10^{13}$ vg/mouse dose of the 1st generation vector (green) compared to other doses and vehicle control. Arrow indicates age at injection. **(b-c)** Plots showing mean bodyweight and aggregate severity score, respectively, for these cohorts after injection. Data presented as mean ± SEM. **(d)** Flattened confocal stack images of the hippocampus CA1 region of wild-type mice injected with $1x10^{13}$ vg/mouse of the 1st generation vector. Tissues were immunolabelled with anti-Myc and anti-MeCP2 antibodies. White arrows indicate transduced cells. Scale bar indicates 20 µm. **(e)** Quantification of cellular levels of native MeCP2 and vector-derived MeCP2 in transduced and non-transduced cells in the hippocampus CA1 region of wild-type mice (n=2 mice; 131 transduced cells and 172 non-transduced cells). Data presented as mean ± SEM and normalised to native MeCP2. **(f)** Frequency distribution of normalised MeCP2 level in transduced and non-transduced cells. # indicates lethality at high dose.

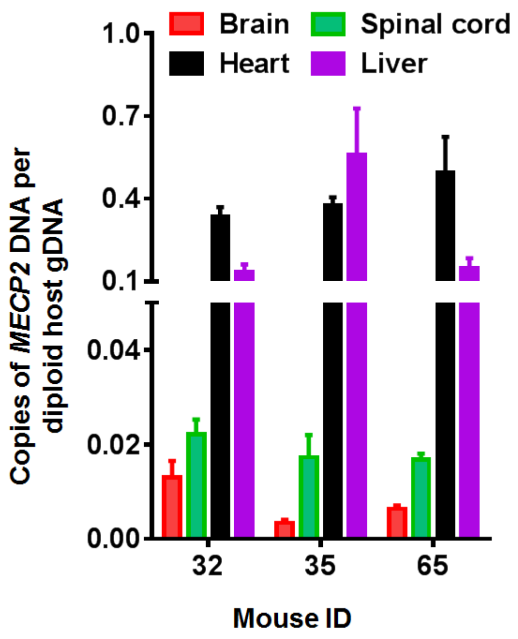

Figure S3. Biodistribution of 1st generation vector after intravenous injection.

Graph showing vector biodistribution in *Mecp2*$^{-/y}$ mice (n=3) as calculated by qPCR. Mice were injected intravenously at 5 weeks of age with 1x10^{12} vg/mouse and samples were taken approximately 22 weeks later. Data were standardised to host genomic DNA and are presented as mean ± SEM.

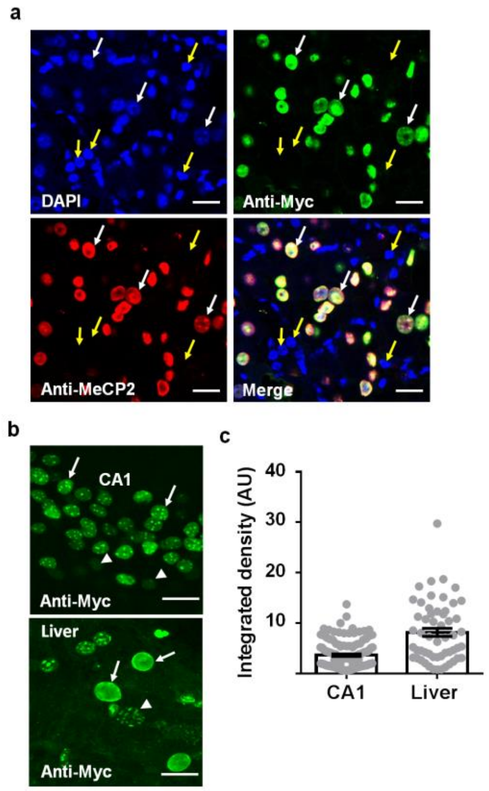

Figure S4. Intravenous injection of 1st generation vector resulted in high level of vector-derived MeCP2 expression in the liver.

(a) Representative confocal images of liver taken from WT mice injected intravenously with 1st generation vector at the dose of $1x10^{13}$ vg/mouse. Sections were immunolabelled with anti-Myc (green), anti-MeCP2 (red) and DAPI nuclear stain (blue). White arrows indicate transduced cells, whereas yellow arrows indicate non-transduced cells. **(b)** Flattened confocal stack images taken from the CA1 region of the hippocampus (top) and from the liver (mice were injected intravenously with $1x10^{13}$ vg/mouse) using the same confocal settings. Arrows indicate nuclei with a high level of vector-derived MeCP2 expression (based on fluorescence intensity of the anti-Myc antibody) and arrowheads indicate nuclei with low expression levels. Scale bar in (a) & (b) = 20 µm. **(c)** measurement of the integrated pixel intensity per nucleus in liver (55 transduced cells and CA1 (131 transduced cells) of the same mice (n = 3 mice). Data presented as mean ± SEM.

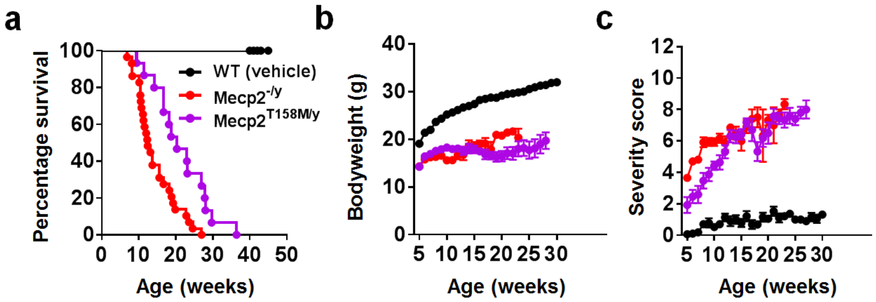

Figure S5. Comparison of *Mecp2$^{T158M/y}$* and *Mecp2$^{-/y}$* mice.

(a) Survival plot for *Mecp2$^{T158M/y}$* mice (n=15) and *Mecp2$^{-/y}$* mice (n=29). **(b-c)** Plots showing no significant differences in mean bodyweight and aggregate severity score, respectively, between *Mecp2$^{T158M/y}$* and *Mecp2$^{-/y}$* mice. Data presented as mean ± SEM.

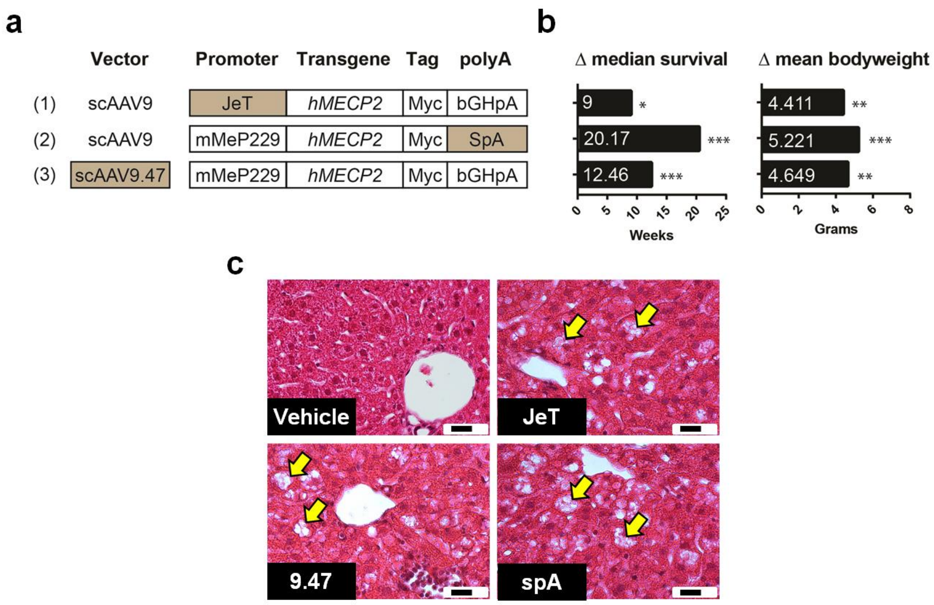

Figure S6. Novel vector design features, efficacy and liver phenotype.

(a) A summary of the design differences for three of the novel vectors described in the text. **(b)** Efficacy of these three novel vectors after intravenous injection of $1x10^{12}$ vg/mouse to 4-5 weeks old *Mecp2*$^{-/y}$ mice, , expressed as increase in median survival relative to the vehicle controls (left; compared using Mantel-Cox test) and mean bodyweight at the age of 11 weeks (right) relative to the vehicle controls (one-way ANOVA with Tukey's post-hoc pairwise comparisons). * $p < 0.05$, ** $p < 0.01$, *** $p < 0.001$. **(c)** Representative H&E-stained liver sections from mice injected with JeT, 9.47 or spA vectors. Arrows indicate vacuolation of hepatocytes; scale bar indicates 20 µm.

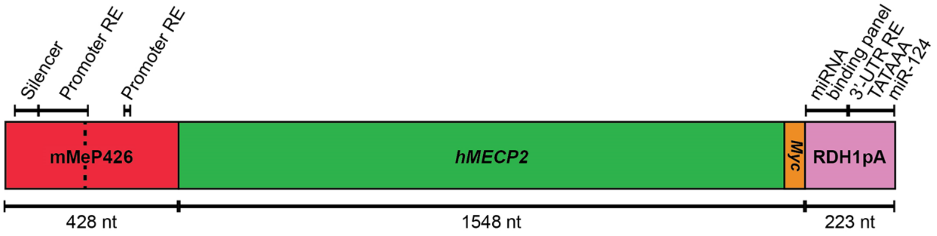

Regulatory element (RE)	Reference
Silencer	Liu & Francke (2006)*
Promoter RE	Adachi et al. (2005); Liu & Francke (2006)*; Liyanage et al. (2013)
miR-22 binding site	Feng et al. (2014)
miR-19 binding site	Jovicic et al. (2013)
miR-132 binding site	Klein et al. (2007)
3'-UTR RE	Coy et al. (1999); Newnham et al. (2010)*; Bagga & D'Antonio (2013)*
TATAAA polyadenylation signal	Coy et al. (1999); Newnham et al. (2010)*; Bagga & D'Antonio (2013)*
miR-124 binding site	Visvanathan et al. (2007); Jovicic et al. (2013)

Figure S7. Design of the 2nd generation vector construct.

Putative regulatory elements (RE) in the extended mMeP426 promoter and endogenous distal 3'-UTR are indicated. The extent of the mMeP229 promoter (used in the 1st generation vector) is indicated by the dashed line. Two non-endogenous cytosine nucleotides precede the ATG start codon. The RDH1pA 3'-UTR consists of several exogenous microRNA (miR) binding sites incorporated as a 'binding panel' adjacent to a portion of the distal endogenous *MECP2* polyadenylation signal and its accompanying regulatory elements. References with an asterisk indicate human *in vitro* studies, not rodent.
