# Supplementary material for: Development of a Novel AAV Gene Therapy Cassette with Improved Safety Features and Efficacy in a Mouse Model of Rett Syndrome
Source: Mol Ther Methods Clin Dev. 2017 Apr 22;5:180–90. doi: 10.1016/j.omtm.2017.04.007 (PMC5423329; doi:10.1016/j.omtm.2017.04.007)
Supplement: Document S2. Article plus Supplemental Information [file mmc2.pdf]

Molecular Therapy
Methods & Clinical Development
Original Article

Development of a Novel AAV Gene Therapy Cassette with Improved Safety Features and Efficacy in a Mouse Model of Rett Syndrome

Kamal K.E. Gadalla,[1,2] Thishnapha Vudhironarit,[1] Ralph D. Hector,[1] Sarah Sinnett,[3,4] Noha G. Bahey,[1,5] Mark E.S. Bailey,[6] Steven J. Gray,[3,4,7] and Stuart R. Cobb[1]

[1]Institute of Neuroscience and Psychology, College of Medical, Veterinary and Life Sciences, University of Glasgow, Glasgow G12 8QQ, UK; [2]Pharmacology Department, Faculty of Medicine, Tanta University, Tanta 31527, Egypt; [3]Gene Therapy Center, University of North Carolina at Chapel Hill, Chapel Hill, NC 27599, USA; [4]Carolina Institute for Developmental Disabilities, University of North Carolina at Chapel Hill, Chapel Hill, NC 27599, USA; [5]Histology Department, Faculty of Medicine, Tanta University, Tanta 31527, Egypt; [6]School of Life Sciences, College of Medical, Veterinary and Life Sciences, University of Glasgow, Glasgow G12 8QQ, UK; [7]Department of Ophthalmology, University of North Carolina at Chapel Hill, Chapel Hill, NC 27514, USA

Rett syndrome (RTT), caused by loss-of-function mutations in the *MECP2* gene, is a neurological disorder characterized by severe impairment of motor and cognitive functions. The aim of this study was to investigate the impact of vector design, dosage, and delivery route on the efficacy and safety of gene augmentation therapy in mouse models of RTT. Our results show that AAV-mediated delivery of *MECP2* to *Mecp2* null mice by systemic administration, and utilizing a minimal endogenous promoter, was associated with a narrow therapeutic window and resulted in liver toxicity at higher doses. Lower doses of this vector significantly extended the survival of mice lacking MeCP2 or expressing a mutant T158M allele but had no impact on RTT-like neurological phenotypes. Modifying vector design by incorporating an extended *Mecp2* promoter and additional regulatory 3′ UTR elements significantly reduced hepatic toxicity after systemic administration. Moreover, direct cerebroventricular injection of this vector into neonatal *Mecp2*-null mice resulted in high brain transduction efficiency, increased survival and body weight, and an amelioration of RTT-like phenotypes. Our results show that controlling levels of MeCP2 expression in the liver is achievable through modification of the expression cassette. However, it also highlights the importance of achieving high brain transduction to impact the RTT-like phenotypes.

INTRODUCTION

Rett syndrome (RTT; OMIM 312750) is a neurological disorder characterized by a constellation of clinical diagnostic and associated features and with overt onset occurring several months postnatally.[1] Typical RTT is almost exclusively caused by de novo germline mutations in the X-linked gene, *MECP2*[2] (as reviewed elsewhere[3,4]). Several mouse models of RTT have been generated that harbor *Mecp2* deletions[5–7] or knocked-in mutations.[8–11] Many of these models recapitulate the principal features that characterize RTT in humans, although there are differences that reflect the phenotypic variability seen in patients.[12–14] Despite the severity of RTT-like phenotypes, genetic reactivation of silenced *Mecp2* in conditional knockout mice resulted in a robust and enduring reversal of phenotypes.[15–17]

This inherent reversibility of the phenotype, added to the lack of obvious targets for pharmacotherapy, makes gene therapy an obvious therapeutic strategy in RTT. However, there are significant challenges to a gene transfer approach, including the requirement to transduce sufficient numbers of neurons in the brain[16] and the avoidance of deleterious overexpression.[18]

Previous attempts at *MECP2* gene transfer using AAV9 vectors were confounded by limited brain transduction efficiency and toxicity,[19,20] while efficacy in other studies using self-complementary adeno-associated virus (AAV) (scAAV)[21] may have been compromised by the use of a construct exceeding the packaging capacity of the vector.

The aim of the present study was to assess the therapeutic impact of dose, route of administration, and expression cassette design in mice modeling RTT. Our results show that modification of the vector design by incorporating more regulatory elements is able to reduce peripheral expression of vector-derived MeCP2 and prevent liver toxicity. We also show that using the same vector design by direct brain injection in mouse neonates resulted in higher brain transduction and improved the RTT-like phenotype.

RESULTS

Dose Escalation with AAV/*MECP2* Revealed a Narrow Therapeutic Window following Systemic Administration

In order to explore the relationship between vector dose and therapeutic benefits, we conducted a dose escalation experiment in which

Received 24 February 2017; accepted 19 April 2017;
http://dx.doi.org/10.1016/j.omtm.2017.04.007.

Correspondence: Stuart Cobb, Institute of Neuroscience and Psychology, College of Medical Veterinary and Life Sciences, West Medical Building, Glasgow, G12 8QQ UK.
E-mail: stuart.cobb@glasgow.ac.uk

This is an open access article under the CC BY license (http://creativecommons.org/licenses/by/4.0/).

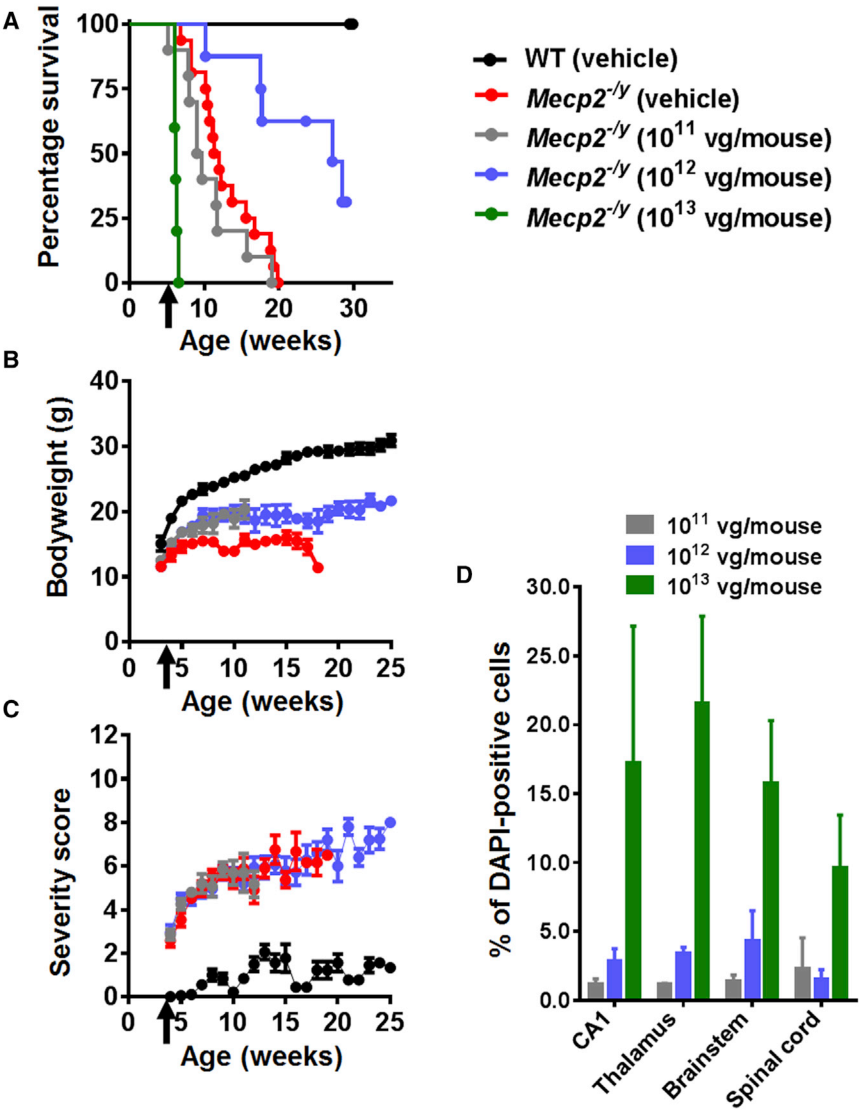

Figure 1. Systemic Delivery of the First-Generation Vector to *Mecp2*$^{-/y}$ Mice Revealed Therapeutic Efficacy and a Narrow Therapeutic Window

(A) Kaplan-Meier survival plot for *Mecp2*$^{-/y}$ mice injected with different doses (1×10^{11} [n = 10], 1×10^{12} [n = 8], and 1×10^{13} [n = 5] vg per mouse] of first-generation vector compared to vehicle-treated animals (WT; n = 9, *Mecp2*$^{-/y}$; n = 16). The median survival period in *Mecp2*$^{-/y}$ mice treated with 1×10^{12} vg per mouse was significantly higher than that in vehicle-treated controls (27.14 versus 11.64 weeks; p = 0.001, Mantel-Cox test). (B and C) Plots showing mean (B) body weight and (C) aggregate severity scores for *Mecp2*$^{-/y}$ mice treated with 1×10^{11} and 1×10^{12} vg per mouse or vehicle. Arrows indicate age at injection; data are presented as mean ± SEM. (D) Dose-dependent transduction efficiency (Myc-positive nuclei as a proportion of DAPI-positive nuclei) across different brain regions. Data are presented as mean ± SEM (n = 3 mice per group). CA1 indicates hippocampal region CA1.

an scAAV2/9 vector was used to deliver a Myc-tagged human *MECP2_e1* cDNA under the control of a short, 229-bp region of the murine *Mecp2* endogenous core promoter (MeP229),[19,22] hereinafter referred to as the "first-generation vector". Juvenile male *Mecp2*$^{-/y}$ and wild-type (WT) mice were injected at the age of 4–5 weeks in the tail vein either with vehicle or with 1×10^{11} (low dose), 1×10^{12} (moderate dose), or 1×10^{13} (high dose) viral genomes (vg) per mouse (dose range, $\sim 1 \times 10^{13}$–1×10^{15} vg/kg). As expected from previous studies of this knockout line,[6,7,15] onset of RTT-like phenotypic signs in control vehicle-treated *Mecp2*$^{-/y}$ mice[15] was observed from 4 to 5 weeks of age, and severity progressively increased until death or censoring of all mice by 20 weeks of age (Figures 1A–1C). *Mecp2*$^{-/y}$ mice treated with the low dose were indistinguishable from vehicle-treated *Mecp2*$^{-/y}$ mice in terms of survival (median survival = 9.36 weeks versus 11.64 weeks, respectively; p = 0.2, Mantel-Cox test,) and severity score (Figures 1A and 1C). However, when measured at 11 weeks (the median survival time for the control vehicle-treated *Mecp2*$^{-/y}$ mice), the mean body weight of the treated *Mecp2*$^{-/y}$ mice was significantly ($p < 0.05$) higher than that of the *Mecp2*$^{-/y}$ vehicle controls (Figure 1B).

In contrast, *Mecp2*$^{-/y}$ mice treated with the moderate dose (1×10^{12}) showed significantly increased survival and body weight compared to the vehicle controls (median survival = 27.3 weeks versus 11.64 weeks; p = 0.001, Mantel-Cox test [Figure 1A]; $p < 0.05$ for mean body weight measured at 11 weeks of age [Figure 1B]). However, there was no difference in the RTT-like phenotype severity score at this dose (Figure 1C). Finally, the cohort receiving the highest dose showed acute toxicity and lethality at 10–15 days post-injection (Figure 1A). Overall, vehicle-treated WT mice differed from *Mecp2*$^{-/y}$ cohorts across all measures (all ps < 0.001).

Patterns of transduction in treated *Mecp2*$^{-/y}$ mice were assessed within the CNS by anti-Myc antibody immunofluorescence labeling (Figure S1), which revealed vector-derived MeCP2 protein expression distributed in a punctate pattern within cell nuclei corresponding to that observed for endogenous MeCP2 in WT mice. Samples from the low-dose cohort revealed low transduction efficiencies across brain regions (0.5% to 1%). The moderate dose resulted in ~3%–5% transduction efficiency, whereas the efficiency for the high dose was 10%–22% (Figure 1D).

In order to measure cellular levels of vector-derived MeCP2 relative to native levels, WT mice were treated with vector as described

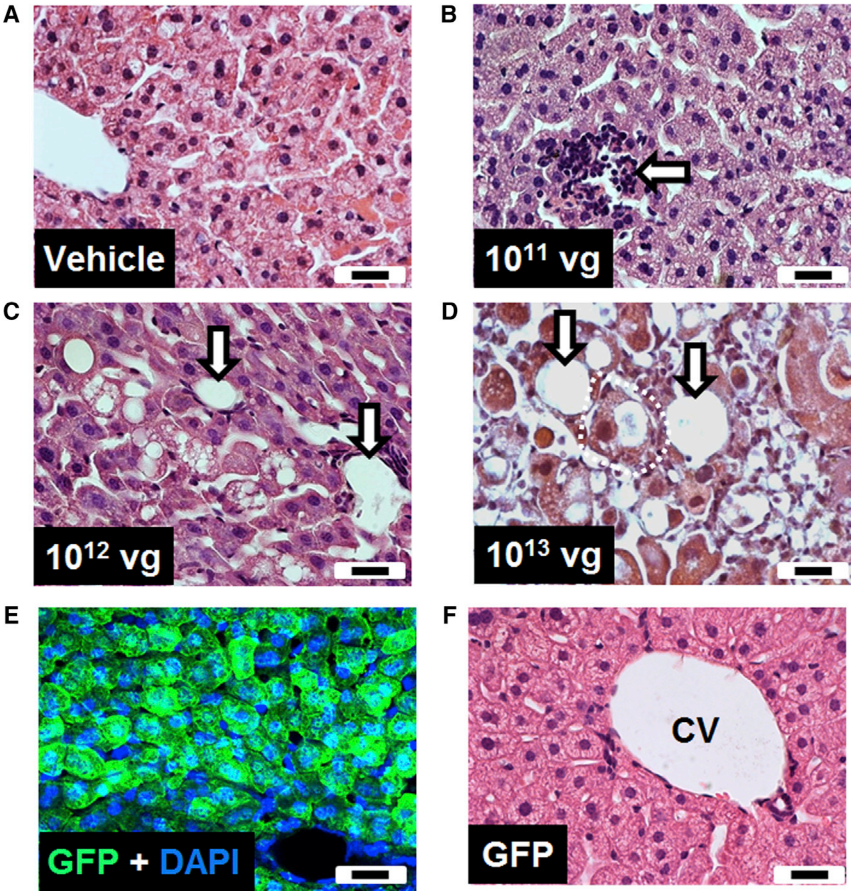

Figure 2. Intravenous Injection of the First-Generation Vector Resulted in Pathological Changes in the Liver

(A–D) Representative H&E-stained liver sections from WT mice injected with (A) vehicle or (B–D) different doses of vector. (E) Liver section from a mouse injected intravenously with a GFP control vector, counterstained with DAPI. (F) Representative H&E-stained liver section from a GFP vector-treated mouse. Arrows indicate mononuclear cell infiltration, vacuolation, and/or loss of hepatocytes. Dashed white line indicates cellular swelling. Scale bars indicate 20 μm. CV, central vein.

earlier. The low and moderate doses were tolerated and had no observable effect on body weight or phenotypic severity score (Figures S2A–S2C). However, WT mice treated with the high dose exhibited the acute toxicity and rapid lethality observed in the knockout mice (Figures S2A–S2C). Quantification of cellular levels of MeCP2 in mice given this high dose revealed that transduced hippocampal pyramidal cells expressed vector-derived MeCP2 at a mean level equivalent to 120% of the endogenous level, resulting in total cellular levels of MeCP2 just over 2-fold higher than normal for these cells (Figures S2D–S2F).

Systemic Delivery of First-Generation Vector Resulted in Liver Toxicity

To further investigate toxic effects encountered after systemic injection of the first-generation vector at high doses, levels of vector-derived MeCP2 expression were tested in a range of peripheral tissues. Bio-distribution of the vector genome in different organs was quantified using qPCR at the end of experiment (Figure S3) and revealed, along with immunohistochemistry, that the proportion of Myc-positive cells in the liver was high (Figure S4). Endogenous MeCP2 levels are known to be much lower in liver cells than in brain neurons[23,24] and are typically below the detection threshold for immunohistochemistry using available antibodies (Figure S4A). However, vector-derived MeCP2 levels in a subset of liver cells (using anti-Myc-immunolabeling) of treated WT mice were found to be higher than MeCP2 levels seen in neurons (Figures S4B and S4C) and were thus ~20 times higher than levels found endogenously in such cells. Myc-positive cells were detected also in the heart, kidney, and other peripheral tissues in treated *Mecp2*$^{-/y}$ mice (data not shown).

Histological investigation of liver sections from mice injected with vehicle or a low dose of the vector showed a largely normal liver structure with occasional areas of mononuclear infiltration (Figures 2A and 2B). In contrast, mice injected with higher doses of the vector showed a dose-dependent increase in pathological features, including cellular destruction and vacuolation, loss of hepatocytes, and mononuclear cell infiltration (Figures 2C and 2D).

To address whether the observed liver pathology was due to the high copy number of viral particles per se or was a consequence of MeCP2 overexpression, we injected mice with a vector driving expression of GFP but otherwise identical to the first-generation vector. Despite detection of widespread GFP expression in the liver (Figure 2E), histological examination of liver sections revealed no evidence of cellular damage or immune cell infiltration (Figure 2F). In addition, no changes in RTT aggregate severity score were observed with this vector (data not shown).

Systemic Administration of First-Generation Vector Improves Survival in *Mecp2*$^{T158M/y}$ Knockin Mice

An important question for gene transfer in RTT is whether the presence of endogenous mutant MeCP2 might reduce the therapeutic effect of vector-derived wild-type MeCP2. Male mice expressing native MeCP2 tagged with GFP as a fusion protein and harboring the common RTT-causing p.T158M mutation,[9] *Mecp2*$^{T158M/y}$, display a phenotype very similar to that of *Mecp2* null mice (Figure S5), but with somewhat enhanced survival (median survival = 20.3 weeks and 12.4 weeks, respectively; $p = 0.0016$, Mantel-Cox test).

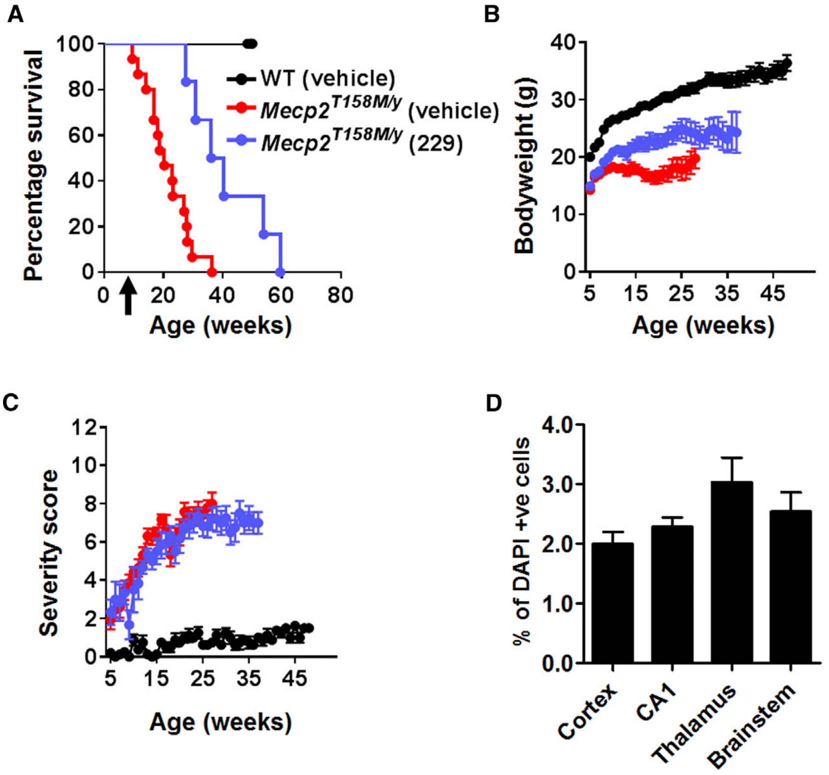

Figure 3. Improved Survival and Body Weight of *Mecp2*$^{T158M/Y}$ Mice after Systemic Delivery of the First-Generation Vector

(A) Survival plot for treated *Mecp2*$^{T158M/y}$ mice. Arrow indicates age at injection. (B and C) Plots of (B) body weight and C) aggregate severity score, respectively, for *Mecp2*$^{T158M/y}$ mice treated with 1 × 10^{12} vg per mouse of first-generation vector and control groups (*Mecp2*$^{T158M/y}$ and WT) treated with vehicle. Data presented as mean ± SEM. (D) Transduction efficiency in the brain of treated mice (Myc-positive nuclei as a proportion of DAPI-positive nuclei; n = 3 mice).

Intravenous delivery of a moderate dose (1 × 10^{12} vg per mouse) of the first-generation vector to 4- to 5-week-old *Mecp2*$^{T158M/y}$ mice resulted in significantly increased survival (Figure 3A; median survival = 38.3 weeks in vector-treated mice versus 20.3 weeks in vehicle-treated mice; p = 0.0019, Mantel-Cox test, n = 8–15 per group). There was a modest increase in body weight in the vector-treated cohort (Figure 3B; $p < 0.05$, one-way ANOVA using data at 20 weeks of age). However, there was no difference in RTT-like aggregate severity score between groups (Figure 3C), consistent with a low brain transduction efficiency (~2%–4%) as revealed by anti-Myc labeling (Figure 3D). Overall, vehicle-treated WT mice differed from *Mecp2*$^{T158M/y}$ cohorts across all measures (all ps < 0.01).

The p.T158M mutation affects the chromatin-binding capacity of MeCP2, leading to loss of the punctate element of MeCP2 labeling in the nucleus (Figure 4A).[9] Immunolabeling of hippocampal neurons from treated *Mecp2*$^{T158M/y}$ mice showed WT patterns of MeCP2 expression, with restored localization to DAPI bright spots, only in transduced (Myc-positive) cells (Figure 4B). This is consistent with vector-derived MeCP2 being able to localize normally to heterochomatin, despite the presence of mutant endogenous MeCP2 protein within the same nucleus.

Development of a Second-Generation Vector that Reduced Liver Toxicity after Systemic Administration

In light of the data described earlier, it was evident that a higher AAV vector dose is required to achieve therapeutically relevant levels of brain transduction after systemic delivery. However, severe toxicity after delivery of high doses of our first-generation cassette necessitated a new design. We tested a range of modifications to the expression cassette and capsid that were predicted to result in lower cellular expression levels and/or reduce liver tropism. This included the use of expression cassettes utilizing (1) an alternative, compact, and, presumably, weaker JeT promoter[25]; (2) a short synthetic polyadenylation (SpA) signal (Figure S6A)[26]; and (3) the original first-generation expression cassette packaged in a scAAV9.47 capsid, which emerged from an in vivo screen for liver de-targeted capsid sequences relative to AAV9.[27,28] Systemic injection of these vectors at the moderate dose (1 × 10^{12} vg per mouse) into 4- to 5-week-old *Mecp2*$^{-/y}$ mice resulted in significantly extended survival and improved body weight, but there was no impact on the RTT-like aggregate severity score (Figure S6B). In summary, none of these modifications resulted in any significant improvements over the first-generation vector ($p > 0.05$ for all measures, ANOVA and Mantel-Cox tests). Importantly, these modified vectors all caused the development of liver pathology similar to that observed with the first-generation vector (as previously shown in Figure 2; Figure S6C).

The rationale for using an endogenous *Mecp2* core promoter fragment (MeP229) in the first-generation vector was that it had been shown largely to recapitulate the endogenous tissue-level pattern of MeCP2 expression.[22] However, this core promoter fragment is missing a number of predicted upstream regulatory elements that may be important in cell-type-specific regulation of MeCP2 expression.[29–31] Therefore, we designed a second-generation vector in which we used an extended promoter fragment (MeP426) incorporating additional promoter regulatory elements and a putative silencer element (Figure S7). We predicted that this might better enable the regulation of vector-derived MeCP2 levels in transduced cells. In addition to the extended promoter, we also incorporated a novel 3′ UTR, consisting of a fragment of the endogenous *MECP2* 3′ UTR together with a selected panel of binding sites for microRNAs (miRNAs) known to be involved in regulation of *Mecp2*[32–35] (Figure S7).

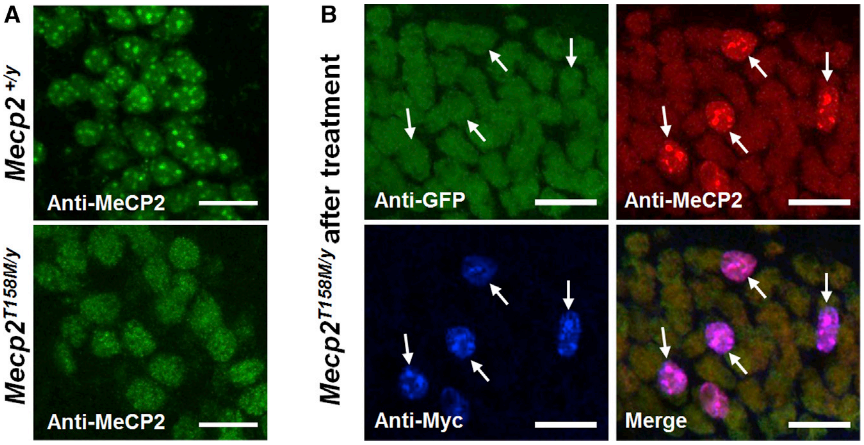

Figure 4. Nuclear Localization of MeCP2 in Untreated and Treated *Mecp2*$^{T158M/Y}$ Mice

Representative confocal images of the CA1 region of the hippocampus. (A) Endogenous MeCP2 exhibits heterochromatin-enriched localization in WT nuclei, while GFP-tagged MeCP2 exhibits decreased heterochromatin localization (i.e., more diffuse labeling) in nuclei from *Mecp2*$^{T158M/y}$ mice. (B) Images demonstrating heterochromatin-enriched localization of vector-derived MeCP2 in nuclei of transduced cells in *Mecp2*$^{T158M/y}$ mice treated with the first-generation vector. White arrows indicate transduced cells (Myc-positive). Scale bars indicate 20 μm.

In order to test the therapeutic efficacy of the second-generation vector, a moderate dose (1×10^{12} vg per mouse) was injected intravenously into 4- to 5-week-old *Mecp2*$^{-/y}$ mice. There was a significant extension of survival in the vector-treated mice compared to the vehicle-treated mice (median survival = 29.9 weeks and 11.6 weeks, respectively; $p < 0.0001$, Mantel-Cox; Figure 5B). There was also significant improvement in body weight at the age of 11 weeks ($p < 0.05$, one-way ANOVA, with Tukey's post hoc pairwise comparison test; Figure 5C). In contrast, there was no effect on RTT-like aggregate severity score (Figure 5D). The second-generation vector, thus, showed no therapeutic advantages over the first-generation vector after systemic delivery (Figures 5B–5D). Again, vehicle-treated WT mice differed from *Mecp2*$^{-/y}$ cohorts across all measures (all $p < 0.001$). In order to compare this vector head-to-head with the first-generation vector in terms of liver safety, mice were injected intravenously with either the first- or the second-generation vector at a dose of 1×10^{12} vg per mouse. These mice were sacrificed after 30 days, and tissues were analyzed for vector-derived MeCP2 expression (using anti-Myc tag antibody) and signs of liver pathology (Figure 6). There was no significant difference in transduction efficiency between vector constructs (Figure 6B), but cellular levels of vector-derived MeCP2 (anti-Myc) in mice treated with first-generation vector were significantly higher than those in mice treated with second-generation vector (Figure 6C; $p < 0.001$, unpaired t test). Analysis of the distribution of cellular MeCP2 expression levels in transduced cells showed that MeCP2 expression was more tightly regulated in mice injected with the second-generation vector (Figure 6D), with fewer cells exhibiting very high expression levels. Moreover, there was none of the disrupted hepatic architecture or vacuolation previously observed with the first-generation vector (Figure 6E). The density of inflammatory foci was significantly higher in liver samples from mice injected with first-generation vector than from those injected with the second-generation vector (Figure 6F).

Neonatal Cerebroventricular Injection of the Second-Generation Vector Improved the RTT-like Aggregate Severity Score

The lack of impact on the phenotype after systemic administration is consistent with the low brain transduction efficiencies observed, as it has been established that phenotype severity and degree of improvement after gene restoration correlate with the proportion of MeCP2-expressing cells in the brain.[16] Therefore, we decided to test the second-generation vector by direct cerebroventricular injection in mouse neonates, a delivery route that is known to afford widespread transgene expression.[19] When delivered at a dose of 1×10^{11} vg per mouse (Figure 7A), there was a pronounced extension in the survival of *Mecp2*$^{-/y}$ mice treated with the second-generation vector in comparison to vehicle-treated mice (median survival = 38.5 and 12.4 weeks, respectively; $p < 0.0001$, Mantel-Cox test; Figure 7B). While there was a negligible effect of vector on body weight (Figure 7C), an important observation was the clear improvement in the RTT-like aggregate severity score compared to that of vehicle-treated *Mecp2*-null mice (Figure 7D; $p < 0.01$ at 11 weeks, one-way ANOVA, with Tukey post hoc pairwise comparison). Vector-derived MeCP2 (revealed by anti-Myc tag immunolabeling) was detectable in all brain regions, with transduction efficiencies across brain regions ranging from ~10%–40% (Figures 7E and 7F). Distribution analysis revealed that the modal cellular MeCP2 level in transduced cells in cortex was approximately twice that of endogenous MeCP2 (consistent with a vector-derived expression level equal to the endogenous level), with some cells expressing higher levels of vector-derived MeCP2 (Figure 7G).

DISCUSSION

The reversal of a wide range of RTT-like phenotypes in mice following the delayed unsilencing of *Mecp2* provides a strong rationale for gene transfer as a therapeutic strategy in RTT.[15,16] There are likely to be a variety of barriers to translational success that will need to be identified and addressed in order to secure optimal outcomes in human clinical trials. In the present study, we identified particular challenges associated with the systemic delivery of a *MECP2*-bearing gene therapy vector in terms of a narrow therapeutic window driven by low brain transduction efficiency and the appearance of peripheral overexpression toxicity upon further dose escalation. However, peripheral overexpression can be reduced by refining the cassette design. We show that direct brain delivery of vector in neonatal mice can achieve therapeutically relevant levels of

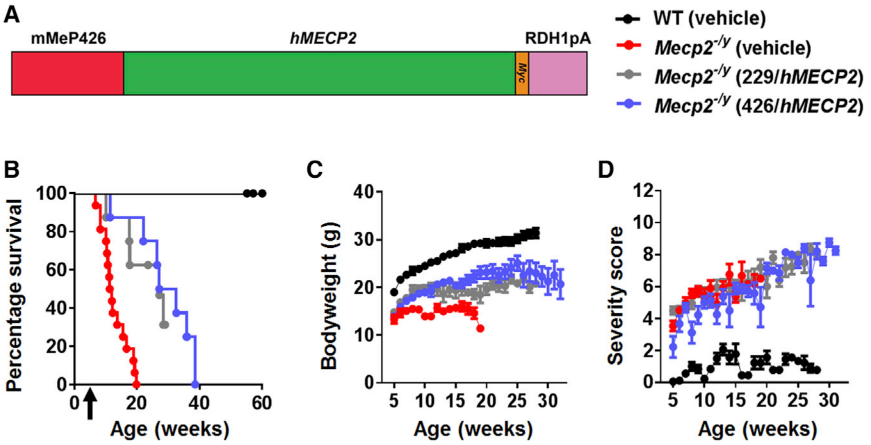

Figure 5. Therapeutic Efficacy of Second-Generation Vector after Systemic Delivery to *Mecp2*$^{-/y}$ Mice

(A) Design features of our second-generation vector summarized (see Results and Figure S7 for details). (B) Survival plot for *Mecp2*$^{-/y}$ mice treated intravenously with 1×10^{12} vg per mouse of the second-generation vector (median survival = 29.9 weeks) or an identical dose of first-generation vector (median survival = 27.1 weeks) or vehicle (median survival = 11.6 weeks). Arrow indicates age at injection. (C and D) Plots showing (C) mean body weight and (D) aggregate severity scores, respectively, of *Mecp2*$^{-/y}$ mice treated as in (B). See also Figure S7.

transduction that result in phenotype amelioration. We also show that the vector has similar effectiveness in mice expressing the most common RTT-causing mutation, suggesting that the presence of existing mutant forms of MeCP2 is unlikely to be an obstacle to translational success. These results are consistent with experiments in transgenic mice expressing both mutant and WT forms of the protein.[36]

Recent attempts to deliver *MECP2* exogenously in mouse models of RTT used widely varying vector doses but are difficult to compare based on additional differences in cassette design and other variables, including viral production, dosing protocol, and phenotype measures.[19–21] In the present study, we used our previously published cassette design (human *MECP2_e1*, under the control of a MeP229 core promoter fragment)[19] to directly investigate the effect of dose in terms of efficacy and safety. A notable finding was the overall lack of efficacy across the range of doses tested in terms of an effect on RTT-like phenotype severity score. This is not due to such phenotypes being inherently resistant to reversal[15,16] but is instead most likely explained by the low levels of brain transgene expression afforded by this route of delivery. In contrast to the phenotype severity score, there was a clear dose-response relationship for survival, with the intermediate dose causing a modest increase in mean body weight and a significant extension in survival. It is not clear whether the survival and body weight effects are due to sufficient (if low) transduction levels in critical brain regions or to expression of MeCP2 in peripheral tissues relevant to mortality. Recent evidence suggests that MeCP2 levels in peripheral tissues can subtly affect body weight,[23] and it is possible that this may indirectly affect survival measures, as we are obliged to use the acute loss of body weight as an endpoint criterion. However, there was a clear divergence in survival between the 10^{11}- and 10^{12}-vg doses without overt differences in mean body weight between groups (Figure 1). Another potential explanation is that we were underestimating levels of transduction efficiency related to survival, based on the sensitivity of our immunohistochemical detection. However, vector biodistribution validation using qPCR was consistent with our measurements, confirming very modest transduction efficiencies following systemic delivery. Only the highest dose tested produced appreciable levels of brain transduction (>10%–20%), and, unfortunately, the severe liver pathology and lethality associated with this dose precluded assessment of the potential for brain-specific therapeutic effects in this situation. Liver cells normally express relatively low levels of MeCP2 compared to neurons,[23] and identical doses of a GFP-expressing vector were not toxic, so the dose-dependent liver pathology is likely to be attributed to the overexpression of vector-derived MeCP2. The difference in the severity score observed in WT mice treated with the moderate dose (which showed no apparent toxicity) and with the high dose (which showed high levels of lethality) can potentially be explained in terms of the cellular levels of MeCP2 that can be tolerated by liver cells—this tolerability threshold may lie between the levels of MeCP2 achieved by the two vector doses.

Previous preclinical RTT gene therapy studies[19–21] have focused on using the *Mecp2*$^{-/y}$ model to screen for vector efficacy and potential toxicity. However, the presence of mutant endogenous MeCP2 could potentially produce a quasi-dominant negative action on the vector-derived MeCP2. We have shown here that, although this knockin line[9] exhibits RTT-like phenotype severity scores similar to those observed in *Mecp2*$^{-/y}$ mice, it also exhibits prolonged survival, thus indicating that the mutant allele may produce MeCP2 with some residual function. Interestingly, AAV-mediated systemic delivery of *MECP2* to these mice resulted in a therapeutic effect similar to that achieved in the *Mecp2*$^{-/y}$ mice treated with the same vector dose. Therefore, we conclude that presence of the mutant protein does not impede the functionality of vector-derived MeCP2. This finding supports the potential translational application of augmentation gene therapy in patients with missense *MECP2* mutations.

Our initial attempts to lower toxic MeCP2 expression and/or reduce liver tropism involved modifications to the expression cassette and capsid. However, the use of putative weaker synthetic promoters and polyadenylation signals was not sufficient to avoid liver toxicity. Surprisingly, the use of an AAV9.47 capsid, which is purported to de-target the liver relative to AAV9,[27,28] resulted in liver pathology similar to that seen with AAV9. Therefore, we focused efforts on a

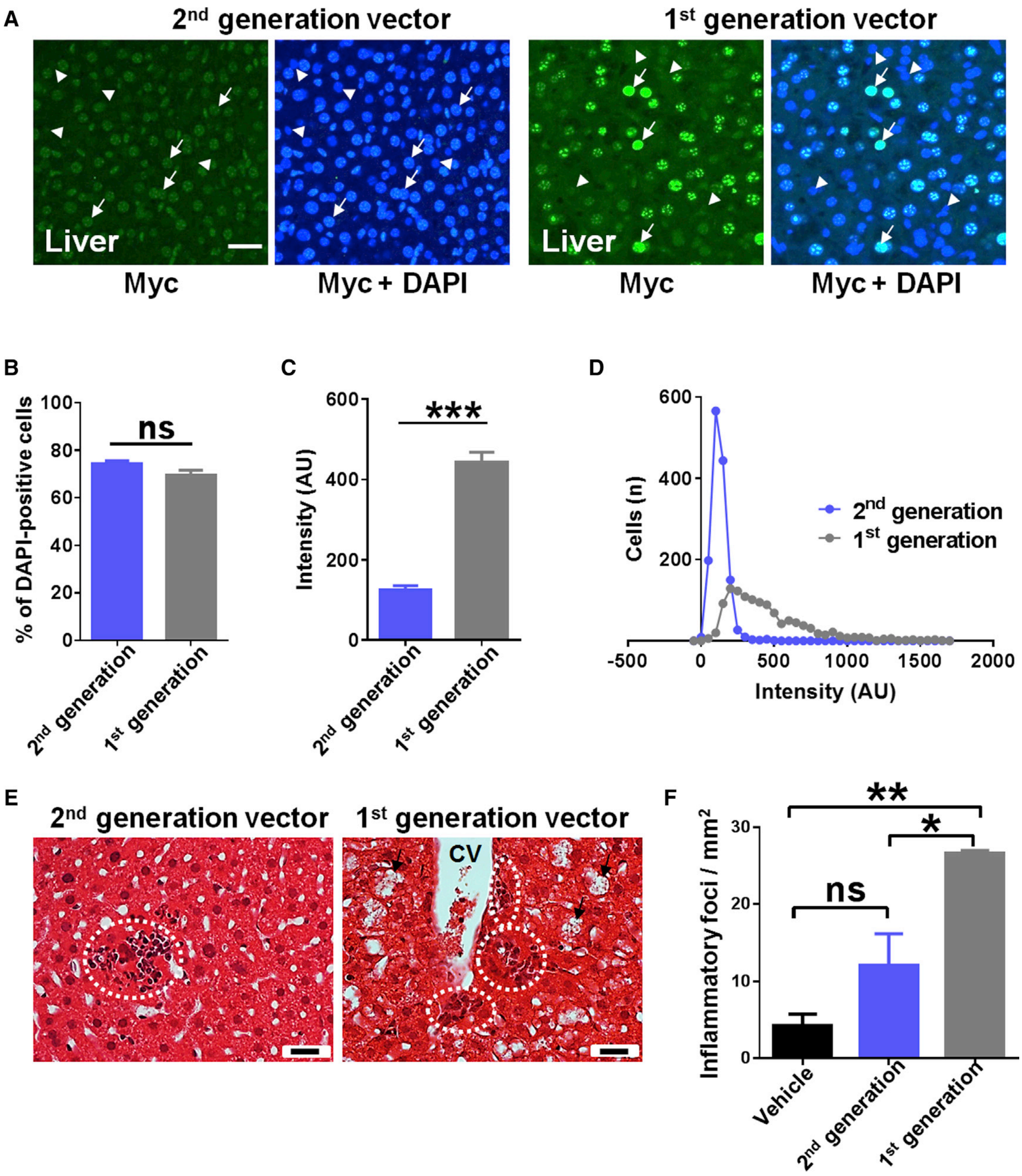

Figure 6. Reduced Expression of Vector-Derived MeCP2 in the Livers of Mice Treated with Second-Generation Vector

(A) Flattened confocal stack images from livers of mice 1 month after being injected intravenously at 5 weeks of age with the second-generation vector or first-generation vector at 1×10^{12} vg per mouse; confocal settings were the same in each case. Tissues were immunolabeled with anti-Myc and DAPI nuclear stain. Arrows indicate transduced cells (Myc-positive), and arrowheads indicate non-transduced cells. (B) Transduction efficiencies in the liver for both vectors. (C) Quantification of cellular levels of vector-derived MeCP2 measured as anti-Myc immunofluorescence in transduced cells in the liver (n = 3 mice, 1,400 transduced cells). Data are presented as mean ± SEM. (D) Frequency distribution of cellular levels of vector-derived MeCP2 in the liver, measured as in (C). (E) Liver sections stained with H&E showing vacuolation of hepatocytes (arrows) and sites of mononuclear cell infiltration (dashed circles). CV, central vein. White scale bar indicates 20 μm. (F) Quantification of density of inflammatory foci in the livers of treated mice (n = 3 per group). Data are presented as mean ± SEM. *$p < 0.05$; **$p < 0.01$; ***$p < 0.001$; ns, not significant.

second-generation vector, whose design was based on the inclusion of endogenous regulatory elements that may better regulate levels of vector-derived MeCP2 in transduced cells. This included the incorporation of an extended endogenous promoter and an endogenous 3′ UTR fragment. Studies analyzing the well-conserved human *MECP2* and mouse *Mecp2* promoter regions indicated the presence

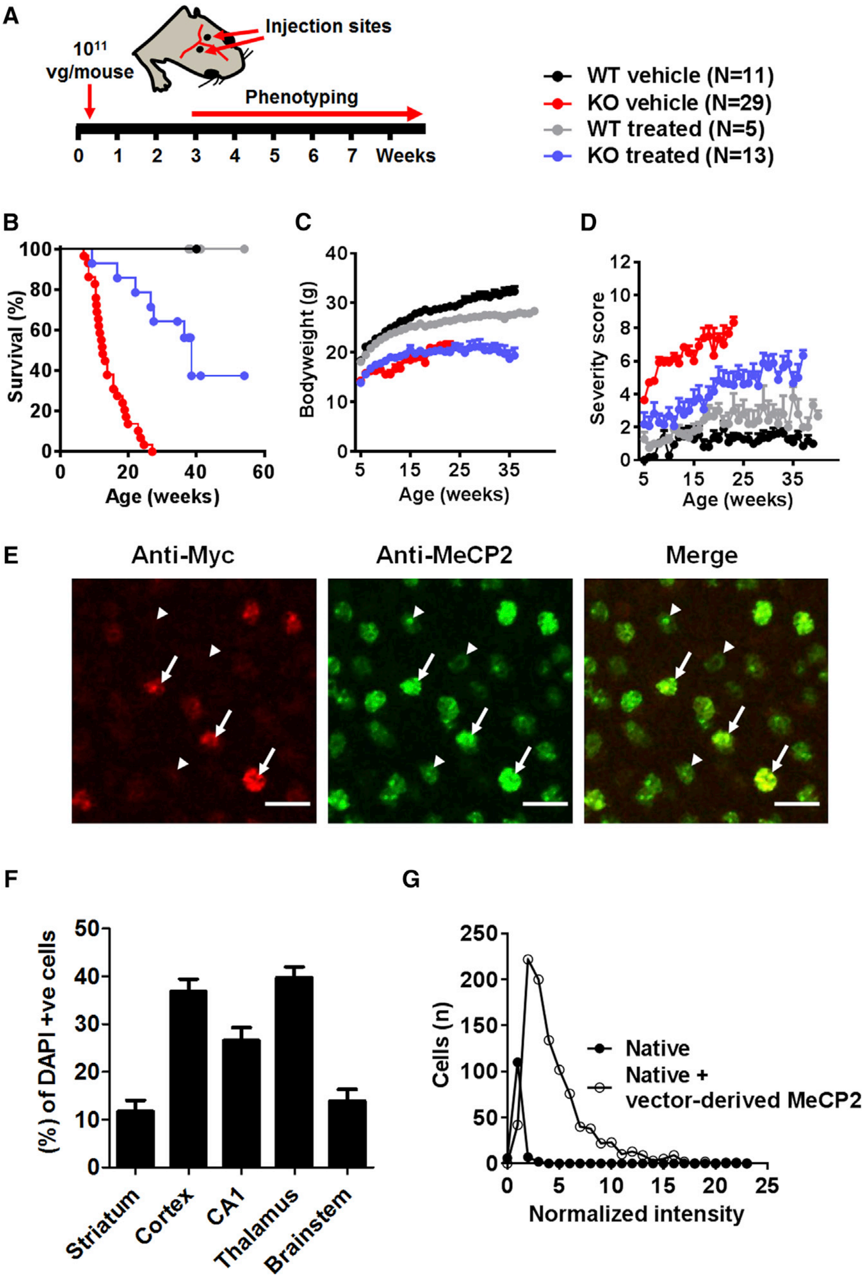

Figure 7. Direct Brain Delivery of Second-Generation Vector to Neonatal *Mecp2*$^{-/y}$ Mice Revealed Therapeutic Efficacy

(A) Experimental design. KO, knockout. (B) Survival plot showing extended survival of neonatally treated *Mecp2*$^{-/y}$ mice (median survival = 38.6 weeks; $p < 0.0001$, Mantel-Cox test) compared with vehicle-treated animals (median survival = 12.4 weeks). (C and D) Plots showing mean (C) body weight and (D) aggregate severity scores, respectively, for the mice shown in (B). (E) Representative confocal images from the cortex of injected wild-type mice. White arrows indicate transduced cells; arrowheads indicate non-transduced cells; scale bars indicate 20 μm. (F) Graph showing transduction efficiency in different brain regions (n = 3 mice). (G) Frequency distribution of MeCP2 levels in transduced and non-transduced ("native") cells in the mouse cortex (n = 3 mice; 954 transduced cells) data presented as mean ± SEM.

of a number of putative regulatory elements within a 1-kb window immediately upstream of the transcription start site.[29–31] Consequently, our extended endogenous promoter (426 bp) in the second-generation vector comprised a putative silencer element at position −274 to −335, with respect to the RefSeq transcription start site (Figure S7).

An endogenous 3′ UTR was also incorporated, containing the distal *MECP2* polyadenylation signal and a number of clustered putative regulatory elements.[37–39] In addition, we performed an analysis of miRNA binding sites in the 3′ UTR of *MECP2*, using a number of bioinformatic tools,[40–42] and incorporated a compact sequence containing the binding sites of three highly conserved miRNAs known to be involved in regulation of MeCP2 in the brain; miR-22,[32] miR-19,[33] and miR-132.[34] Combined, these modifications significantly reduced MeCP2 expression in the liver, with subsequent reduction of the hepatotoxicity encountered with the first-generation vector. The relative importance of different modifications (elements within the extended promoter and novel 3′ UTR) was not investigated. However, the efficacy of both vectors after systemic injection of moderate doses was not significantly different. The important advantage of the second-generation vector is the lack of prominent liver pathology at a dose that provides some therapeutic benefit (i.e., 1×10^{12} vg per mouse). The improved survival after systemic injection, despite low brain transduction efficiency, could be due to restoration of MeCP2 levels in sufficiently numerous critical cells in the brain or due to restoration in important peripheral tissues. Targeting more cells in the brain through direct brain injection in mouse neonates, along with potentially greater impact via earlier intervention, led to pronounced survival enhancement at a dose (1×10^{11} vg per mouse) approximately equivalent to the 10^{12} systemic dose. Delivery by this direct brain injection route was associated with an improvement in body weight but, importantly, also with an improvement in RTT-like phenotype score. The improvement was not as profound as that reported in genetic reversal experiments,[16] and this is likely to be due to the combined effects of (1) the relative inefficiency of MeCP2 re-expression across the brain (10%–40%),

compared to genetic reversal experiments (up to 90%), and (2) the possible deleterious counteracting effects of overexpressing MeCP2 in a proportion of transduced cells. Analysis of MeCP2 levels, indeed, indicates a significant pool of cells overexpressing MeCP2, presumably transduced with multiple copies of vector delivering *MECP2*. This may also account for the slightly elevated severity score in vector-treated WT mice (Figure 7D) in the form of mild hindlimb clasping. We cannot rule out very subtle consequences of MeCP2 overexpression that may be revealed by fine-grained behavioral testing. Overall, the proof-of-concept experiments involving direct brain delivery in neonatal mice suggest that, if transduction efficiency across the brain can reach sufficiently high levels, then a behavioral improvement is conferred by this vector design.

Conclusions

The results of the present study highlight the challenges associated with both systemic and direct brain delivery of *MECP2.* The findings suggest that achieving widespread brain expression, while at the same time maintaining cell-type appropriate control of MeCP2 levels, will be essential requirements for the successful development of a translational therapy. The development of expression cassettes capable of producing effective and sub-toxic levels of MeCP2 may overcome issues of cellular overexpression and enable direct delivery via the cerebrospinal fluid compartment. While AAV9 appears to be insufficiently efficient in terms of brain transduction after systemic delivery of *MECP2* to achieve the desired therapeutic benefit, combining the safer second-generation cassette together with capsids with improved brain penetrance[43] may effectively pair effective CNS gene transfer with safe levels of peripheral MeCP2 transgene expression. Such a combination would hold enhanced translational promise.

MATERIALS AND METHODS

Animals

All experiments were carried out in accordance with the European Communities Council Directive (86/609/EEC) and with the terms of a project license under the UK Scientific Procedures Act (1986). The *Mecp2* null, *Mecp2*$^{tm1.1Bird}$, and *Mecp2*^{T158M} mice, originally provided as a kind gift from Professor Adrian Bird, were maintained on a C57BL/6 background. Animals were maintained on 12-hr:12-hr light/dark cycles with free access to normal mouse food. Mice were genotyped as described previously.[9,15]

Viral Vector Preparation

Recombinant AAV vector particles were generated at the University of North Carolina (UNC) Gene Therapy Center Vector Core facility. The scAAV particles (AAV2 ITR [inverted terminal repeat]-flanked genomes packaged into AAV9 or AAV9.47 serotype capsids) were produced from suspension HEK293 cells transfected using polyethyleneimine (Polysciences) with helper plasmids (pXX6-80 and pGSK2/9) and a plasmid containing the appropriate ITR-flanked transgene construct. All MeCP2-expressing constructs utilized the human *MECP2_e1* coding region with a C-terminal Myc epitope tag unless stated otherwise. Virus production was performed as previously described,[44] and the vectors were prepared in a final formulation of high-salt PBS (containing 350 mmol/L total NaCl) supplemented with 5% sorbitol.

scAAV Vector Injection and Mouse Phenotyping

Frozen scAAV9 viral particle aliquots were thawed and diluted to 100 μL in PBS/350 mmol/L NaCl containing 5% sorbitol. Control injections were made using the same diluent lacking vector ("vehicle control"). For direct brain injection into mouse neonates, littermates were sexed at birth, and direct bilateral injections of virus (3 μL per site) were delivered into the neuropil of unanesthetized males at postnatal day (P)0–P3, as described previously.[19] The injected pups were returned to the home cage containing their non-injected female littermates. Genotyping was carried out at 3 weeks, at which time phenotyping was initiated. For injection into juvenile male mice, injections were made via the tail vein at 4–5 weeks of age. Following injection, all mice were weighed weekly. Phenotyping was carried out, blind to genotype and treatment, twice a week. Mice were scored on an aggregate severity scale using an established protocol (mice were scored for RTT-like phenotypes comprising mobility, gait, breathing, hindlimb clasping, tremor, and general condition).[15,16,19,21] For survival analysis, mice were censored after natural death or if body weight losses exceeded 20% of peak body weight.

Vector Biodistribution Analysis

For these analyses, mice were sacrificed, blood was collected transcardially, and organs were harvested for DNA purification. Genomic DNA was recovered from tissues using the DNeasy Blood and Tissue Kit (QIAGEN). A Qiacube (QIAGEN) was used to carry out automated purifications. Genomic qPCR reactions and analysis were performed on a Roche Lightcycler 480, following the manufacturer's instructions. For the quantification of vector biodistribution, the amount of vector genome present in each sample was standardized against an amplicon from a single-copy mouse gene, Lmnb2, amplified from genomic DNA. Lmnb2 primers and "universal" *MECP2* primers (that amplify mouse and human *MECP2*) were published previously.[19,45]

Immunohistochemistry

Mice were anesthetized with pentobarbitone (50 mg, intraperitoneally) and transcardially perfused with 4% paraformaldehyde (0.1 mol/L PBS). A vibrating microtome (Leica VT1200) was used to obtain 80-μm sections of brain, spinal cord, and liver. Sections were dehydrated by incubation in 50% ethanol in distilled water (v/v) for 30 min and then were washed three times in 0.3 mol/L PBS, followed by incubation in 10 mM sodium citrate (pH 6, 85°C, 30 min) for antigen retrieval. Sections were then incubated in the blocking solution (5% normal goat serum in 0.3mol/L PBS with 0.3% Triton X-100) for 1 hr at room temperature. Samples then were incubated for 48 hr on a shaker at 4°C with the following primary antibodies: rabbit anti-Myc (Abcam, ab9106), mouse monoclonal anti-MeCP2 (Sigma, WH0004204M1), and chicken anti-GFP (Abcam, ab13970). The primary antibodies were then washed off (3× 0.3 mol/L PBST), and secondary antibodies were applied to the sections overnight at 4°C: Alexa Fluor 488 goat anti-mouse/rabbit (Invitrogen; 1/500), Alexa Fluor 546 goat anti-mouse/rabbit (Invitrogen; 1/500), Alexa Fluor 649,

goat anti-mouse (Jackson ImmunoResearch Laboratories, 112-495-003JIR). Finally, sections were incubated with DAPI nuclear stain (Sigma; 1/1,000) for 30 min at room temperature before mounting with Vectashield (Vector Laboratories).

H&E Staining

Liver samples were rinsed with 0.1 mol/L PBS and then dehydrated through ascending grades of ethanol, and they were then cleared in amyl acetate using an automated tissue processor. Specimens were embedded in Paraplast, and sections (10 μm thick) were collected on APES (aminopropyltriethoxysilane)-coated slides and dried overnight in the oven at 37°C. Sections were then deparaffinized through two changes of Histo-Clear (Agar Scientific) for 15 min and rehydrated through descending grades of alcohol (100%, 90%, and 70%). The sections were stained with Mayer's hematoxylin for 8 min and then rinsed using tap water. The nuclei were stained blue by placing the slides into Scott's solution for 1 min and were then rinsed using tap water. Sections were then stained with 1% eosin for 2 min and washed by water. Finally, the sections were dehydrated through ascending grades of alcohol and Histo-Clear before being mounted with DPX. Images were captured using an AxioCam MRc (Zeiss) mounted on a light microscope (Zeiss).

Image Analysis

Analysis of expression patterns, transduction efficiency, and quantification of vector-derived MeCP2 levels within nuclei was carried out on image stacks captured using a Zeiss LSM710 or Zeiss Axiovert LSM510 laser confocal microscope (Zeiss). The z series were taken at 1-μm intervals through the section of interest using a 40× objective. To account for antibodies' penetrability, stack images were taken close to the surface of sections to a maximum depth of 20 μm. To estimate transduction efficiency, images were captured as described earlier, and the ratio of Myc-immunopositive nuclei to DAPI-stained nuclei was calculated for random fields (n = 12 images per region:4 images from each of three mice) from sections of hippocampus (CA1 region), layer 5 of primary motor cortex, thalamus, hypothalamus, brain stem, and striatum. To quantify levels of vector-derived MeCP2 per nucleus in WT mice, confocal stacks (20 μm thick) were obtained as described earlier, and ImageJ software (http://rsbweb.nih.gov/ij/) was used to determine mean MeCP2-channel fluorescence intensity within transduced (Myc +ve) and non-transduced (Myc −ve) cells. Fluorescence in the DAPI channel was used to define the nuclear boundary.

Statistical Analysis

Tests for differences between treatment groups were carried out in GraphPad PRISM using one-way ANOVA, Student's t test, and the Mantel-Cox test (survival curves), as appropriate. $p < 0.05$ was used to define statistical significance. In multi-group comparisons, multiple testing correction for pairwise tests among groups was applied using Tukey's post hoc analysis.

SUPPLEMENTAL INFORMATION

Supplemental Information includes seven figures and can be found with this article online at http://dx.doi.org/10.1016/j.omtm.2017.04.007.

AUTHOR CONTRIBUTIONS

Conceptualization, S.R.C., M.E.S.B., and S.J.G.; Methodology, S.R.C., K.K.E.G., N.G.B., and R.D.H.; Investigation, K.K.E.G., T.V., S.S., N.G.B., and R.D.H.; Writing – Original Draft, S.R.C., K.K.E.G., R.D.H., M.E.S.B., and S.J.G.; Funding Acquisition, S.R.C., S.J.G., M.E.S.B., and K.K.E.G.; Resources, R.D.H. and S.S.; Supervision, S.R.C. and M.E.S.B.

CONFLICTS OF INTEREST

S.J.G. declares a conflict of interest with Asklepios Biopharma, from which he has received patent royalties for intellectual property that is not used in this study.

ACKNOWLEDGMENTS

This work was funded by grants from the Rett Syndrome Research Trust (to S.R.C., M.E.S.B., and S.J.G.), the Chief Scientist Office (ETM/334 to S.R.C. and M.E.S.B.) and the NIH (4T32HD040127-15 to S.S.). We are also grateful to the Rett Syndrome Association Scotland, the R.S. MacDonald Charitable Trust, the Stoneygate Trust, and the Rosetrees Trust for additional support (to S.R.C., M.E.S.B., and K.K.E.G.). T.V. received a Thailand government studentship. Indirect administrative support for S.J.G. was provided by Research to Prevent Blindness to the UNC-CH Department of Ophthalmology. The authors thank John Craig at Glasgow University and Daphne Chen, Clifford Heindel, and Violeta Zaric at UNC for their technical assistance. We also thank the B. Kaspar and G. Mandel labs and other members of the Rett Syndrome Research Trust Gene Therapy Consortium for helpful discussions.

REFERENCES

1. Neul, J.L., Kaufmann, W.E., Glaze, D.G., Christodoulou, J., Clarke, A.J., Bahi-Buisson, N., Leonard, H., Bailey, M.E., Schanen, N.C., Zappella, M., et al.; RettSearch Consortium (2010). Rett syndrome: revised diagnostic criteria and nomenclature. Ann. Neurol. *68*, 944–950.
2. Amir, R.E., Van den Veyver, I.B., Wan, M., Tran, C.Q., Francke, U., and Zoghbi, H.Y. (1999). Rett syndrome is caused by mutations in X-linked MECP2, encoding methyl-CpG-binding protein 2. Nat. Genet. *23*, 185–188.
3. Bienvenu, T., and Chelly, J. (2006). Molecular genetics of Rett syndrome: when DNA methylation goes unrecognized. Nat. Rev. Genet. *7*, 415–426.
4. Lyst, M.J., and Bird, A. (2015). Rett syndrome: a complex disorder with simple roots. Nat. Rev. Genet. *16*, 261–275.
5. Chen, R.Z., Akbarian, S., Tudor, M., and Jaenisch, R. (2001). Deficiency of methyl-CpG binding protein-2 in CNS neurons results in a Rett-like phenotype in mice. Nat. Genet. *27*, 327–331.
6. Guy, J., Hendrich, B., Holmes, M., Martin, J.E., and Bird, A. (2001). A mouse Mecp2-null mutation causes neurological symptoms that mimic Rett syndrome. Nat. Genet. *27*, 322–326.
7. Shahbazian, M., Young, J., Yuva-Paylor, L., Spencer, C., Antalffy, B., Noebels, J., Armstrong, D., Paylor, R., and Zoghbi, H. (2002). Mice with truncated MeCP2 recapitulate many Rett syndrome features and display hyperacetylation of histone H3. Neuron *35*, 243–254.
8. Goffin, D., Allen, M., Zhang, L., Amorim, M., Wang, I.T., Reyes, A.R., Mercado-Berton, A., Ong, C., Cohen, S., Hu, L., et al. (2011). Rett syndrome mutation MeCP2 T158A disrupts DNA binding, protein stability and ERP responses. Nat. Neurosci. *15*, 274–283.
9. Brown, K., Selfridge, J., Lagger, S., Connelly, J., De Sousa, D., Kerr, A., Webb, S., Guy, J., Merusi, C., Koerner, M.V., and Bird, A. (2016). The molecular basis of variable

phenotypic severity among common missense mutations causing Rett syndrome. Hum. Mol. Genet. *25*, 558–570.

10. Lyst, M.J., Ekiert, R., Ebert, D.H., Merusi, C., Nowak, J., Selfridge, J., Guy, J., Kastan, N.R., Robinson, N.D., de Lima Alves, F., et al. (2013). Rett syndrome mutations abolish the interaction of MeCP2 with the NCoR/SMRT co-repressor. Nat. Neurosci. *16*, 898–902.
11. Pitcher, M.R., Herrera, J.A., Buffington, S.A., Kochukov, M.Y., Merritt, J.K., Fisher, A.R., Schanen, N.C., Costa-Mattioli, M., and Neul, J.L. (2015). Rett syndrome like phenotypes in the R255X Mecp2 mutant mouse are rescued by MECP2 transgene. Hum. Mol. Genet. *24*, 2662–2672.
12. Archer, H., Evans, J., Leonard, H., Colvin, L., Ravine, D., Christodoulou, J., Williamson, S., Charman, T., Bailey, M.E., Sampson, J., et al. (2007). Correlation between clinical severity in patients with Rett syndrome with a p.R168X or p.T158M MECP2 mutation, and the direction and degree of skewing of X-chromosome inactivation. J. Med. Genet. *44*, 148–152.
13. Ghosh, R.P., Horowitz-Scherer, R.A., Nikitina, T., Gierasch, L.M., and Woodcock, C.L. (2008). Rett syndrome-causing mutations in human MeCP2 result in diverse structural changes that impact folding and DNA interactions. J. Biol. Chem. *283*, 20523–20534.
14. Leonard, H., Cobb, S., and Downs, J. (2016). Clinical and biological progress over 50 years in Rett syndrome. Nat. Rev. Neurol. *13*, 37–51.
15. Guy, J., Gan, J., Selfridge, J., Cobb, S., and Bird, A. (2007). Reversal of neurological defects in a mouse model of Rett syndrome. Science *315*, 1143–1147.
16. Robinson, L., Guy, J., McKay, L., Brockett, E., Spike, R.C., Selfridge, J., De Sousa, D., Merusi, C., Riedel, G., Bird, A., and Cobb, S.R. (2012). Morphological and functional reversal of phenotypes in a mouse model of Rett syndrome. Brain *135*, 2699–2710.
17. Jugloff, D.G., Vandamme, K., Logan, R., Visanji, N.P., Brotchie, J.M., and Eubanks, J.H. (2008). Targeted delivery of an Mecp2 transgene to forebrain neurons improves the behavior of female Mecp2-deficient mice. Hum. Mol. Genet. *17*, 1386–1396.
18. Gadalla, K.K.E., Ross, P.D., Hector, R.D., Bahey, N.G., Bailey, M.E.S., and Cobb, S.R. (2015). Gene therapy for Rett syndrome: prospects and challenges. Future Neurol. *10*, 467–484.
19. Gadalla, K.K., Bailey, M.E., Spike, R.C., Ross, P.D., Woodard, K.T., Kalburgi, S.N., Bachaboina, L., Deng, J.V., West, A.E., Samulski, R.J., et al. (2013). Improved survival and reduced phenotypic severity following AAV9/MECP2 gene transfer to neonatal and juvenile male Mecp2 knockout mice. Mol. Ther. *21*, 18–30.
20. Matagne, V., Ehinger, Y., Saidi, L., Borges-Correia, A., Barkats, M., Bartoli, M., Villard, L., and Roux, J.C. (2017). A codon-optimized Mecp2 transgene corrects breathing deficits and improves survival in a mouse model of Rett syndrome. Neurobiol. Dis. *99*, 1–11.
21. Garg, S.K., Lioy, D.T., Cheval, H., McGann, J.C., Bissonnette, J.M., Murtha, M.J., Foust, K.D., Kaspar, B.K., Bird, A., and Mandel, G. (2013). Systemic delivery of MeCP2 rescues behavioral and cellular deficits in female mouse models of Rett syndrome. J. Neurosci. *33*, 13612–13620.
22. Gray, S.J., Foti, S.B., Schwartz, J.W., Bachaboina, L., Taylor-Blake, B., Coleman, J., Ehlers, M.D., Zylka, M.J., McCown, T.J., and Samulski, R.J. (2011). Optimizing promoters for recombinant adeno-associated virus-mediated gene expression in the peripheral and central nervous system using self-complementary vectors. Hum. Gene Ther. *22*, 1143–1153.
23. Ross, P.D., Guy, J., Selfridge, J., Kamal, B., Bahey, N., Tanner, K.E., Gillingwater, T.H., Jones, R.A., Loughrey, C.M., McCarroll, C.S., et al. (2016). Exclusive expression of MeCP2 in the nervous system distinguishes between brain and peripheral Rett syndrome-like phenotypes. Hum. Mol. Genet. *25*, 4389–4404.
24. Skene, P.J., Illingworth, R.S., Webb, S., Kerr, A.R., James, K.D., Turner, D.J., Andrews, R., and Bird, A.P. (2010). Neuronal MeCP2 is expressed at near histone-octamer levels and globally alters the chromatin state. Mol. Cell *37*, 457–468.
25. Tornøe, J., Kusk, P., Johansen, T.E., and Jensen, P.R. (2002). Generation of a synthetic mammalian promoter library by modification of sequences spacing transcription factor binding sites. Gene *297*, 21–32.
26. Levitt, N., Briggs, D., Gil, A., and Proudfoot, N.J. (1989). Definition of an efficient synthetic poly(A) site. Genes Dev. *3*, 1019–1025.
27. Pulicherla, N., Shen, S., Yadav, S., Debbink, K., Govindasamy, L., Agbandje-McKenna, M., and Asokan, A. (2011). Engineering liver-detargeted AAV9 vectors for cardiac and musculoskeletal gene transfer. Mol. Ther. *19*, 1070–1078.
28. Karumuthil-Melethil, S., Nagabhushan Kalburgi, S., Thompson, P., Tropak, M., Kaytor, M.D., Keimel, J.G., Mark, B.L., Mahuran, D., Walia, J.S., and Gray, S.J. (2016). Novel vector design and hexosaminidase variant enabling self-complementary adeno-associated virus for the treatment of Tay-Sachs disease. Hum. Gene Ther. *27*, 509–521.
29. Liu, J., and Francke, U. (2006). Identification of *cis*-regulatory elements for MECP2 expression. Hum. Mol. Genet. *15*, 1769–1782.
30. Adachi, M., Keefer, E.W., and Jones, F.S. (2005). A segment of the Mecp2 promoter is sufficient to drive expression in neurons. Hum. Mol. Genet. *14*, 3709–3722.
31. Liyanage, V.R., Zachariah, R.M., and Rastegar, M. (2013). Decitabine alters the expression of Mecp2 isoforms via dynamic DNA methylation at the Mecp2 regulatory elements in neural stem cells. Mol. Autism *4*, 46.
32. Feng, Y., Huang, W., Wani, M., Yu, X., and Ashraf, M. (2014). Ischemic preconditioning potentiates the protective effect of stem cells through secretion of exosomes by targeting Mecp2 via miR-22. PLoS ONE *9*, e88685.
33. Jovičić, A., Roshan, R., Moisoi, N., Pradervand, S., Moser, R., Pillai, B., and Luthi-Carter, R. (2013). Comprehensive expression analyses of neural cell-type-specific miRNAs identify new determinants of the specification and maintenance of neuronal phenotypes. J. Neurosci. *33*, 5127–5137.
34. Klein, M.E., Lioy, D.T., Ma, L., Impey, S., Mandel, G., and Goodman, R.H. (2007). Homeostatic regulation of MeCP2 expression by a CREB-induced microRNA. Nat. Neurosci. *10*, 1513–1514.
35. Visvanathan, J., Lee, S., Lee, B., Lee, J.W., and Lee, S.K. (2007). The microRNA miR-124 antagonizes the anti-neural REST/SCP1 pathway during embryonic CNS development. Genes Dev. *21*, 744–749.
36. Heckman, L.D., Chahrour, M.H., and Zoghbi, H.Y. (2014). Rett-causing mutations reveal two domains critical for MeCP2 function and for toxicity in *MECP2* duplication syndrome mice. eLife *3*, e02676.
37. Coy, J.F., Sedlacek, Z., Bächner, D., Delius, H., and Poustka, A. (1999). A complex pattern of evolutionary conservation and alternative polyadenylation within the long 3″-untranslated region of the methyl-CpG-binding protein 2 gene (MeCP2) suggests a regulatory role in gene expression. Hum. Mol. Genet. *8*, 1253–1262.
38. Bagga, J.S., and D'Antonio, L.A. (2013). Role of conserved *cis*-regulatory elements in the post-transcriptional regulation of the human MECP2 gene involved in autism. Hum. Genomics *7*, 19.
39. Newnham, C.M., Hall-Pogar, T., Liang, S., Wu, J., Tian, B., Hu, J., and Lutz, C.S. (2010). Alternative polyadenylation of MeCP2: Influence of cis-acting elements and trans-acting factors. RNA Biol. *7*, 361–372.
40. Lewis, B.P., Burge, C.B., and Bartel, D.P. (2005). Conserved seed pairing, often flanked by adenosines, indicates that thousands of human genes are microRNA targets. Cell *120*, 15–20.
41. Vorozheykin, P.S., and Titov, I.I. (2015). [Web server for prediction of miRNAs and their precursors and binding sites]. Mol. Biol. (Mosk.) *49*, 846–853.
42. Rehmsmeier, M., Steffen, P., Hochsmann, M., and Giegerich, R. (2004). Fast and effective prediction of microRNA/target duplexes. RNA *10*, 1507–1517.
43. Deverman, B.E., Pravdo, P.L., Simpson, B.P., Kumar, S.R., Chan, K.Y., Banerjee, A., Wu, W.L., Yang, B., Huber, N., Pasca, S.P., and Gradinaru, V. (2016). Cre-dependent selection yields AAV variants for widespread gene transfer to the adult brain. Nat. Biotechnol. *34*, 204–209.
44. Clément, N., and Grieger, J.C. (2016). Manufacturing of recombinant adeno-associated viral vectors for clinical trials. Mol. Ther. Methods Clin. Dev. *3*, 16002.
45. Gray, S.J., Blake, B.L., Criswell, H.E., Nicolson, S.C., Samulski, R.J., McCown, T.J., and Li, W. (2010). Directed evolution of a novel adeno-associated virus (AAV) vector that crosses the seizure-compromised blood-brain barrier (BBB). Mol. Ther. *18*, 570–578.

OMTM, Volume *5*

Supplemental Information

Development of a Novel AAV Gene Therapy Cassette with Improved Safety Features and Efficacy in a Mouse Model of Rett Syndrome

Kamal K.E. Gadalla, Thishnapha Vudhironarit, Ralph D. Hector, Sarah Sinnett, Noha G. Bahey, Mark E.S. Bailey, Steven J. Gray, and Stuart R. Cobb

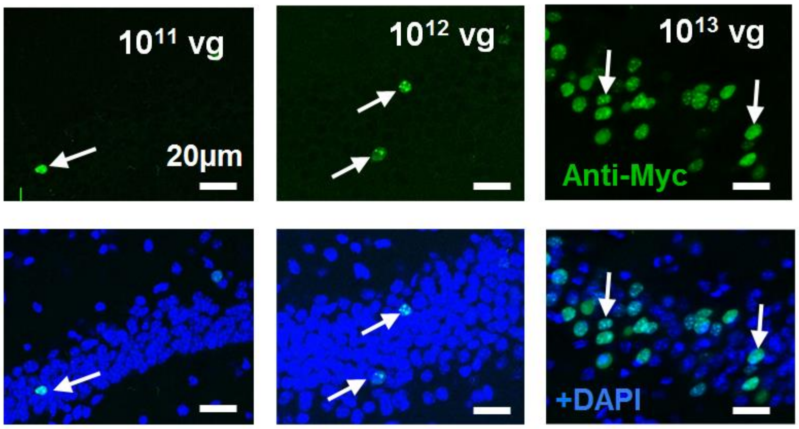

Figure S1. Expression of vector-derived MeCP2 in the brain after intravenous injection of the 1st generation vector.

Representative confocal micrographs showing transgene expression in the hippocampal CA1 region in *Mecp2*$^{-/y}$ mice treated intravenously with 1x10^{11},1x10^{12} and 1x10^{13} vg/mouse of the 1st generation vector (as revealed by anti-Myc tag immunolabelling). Arrows denote transduced cells and the lower panel shows co-localisation with DAPI. Scale bar = 20 µm

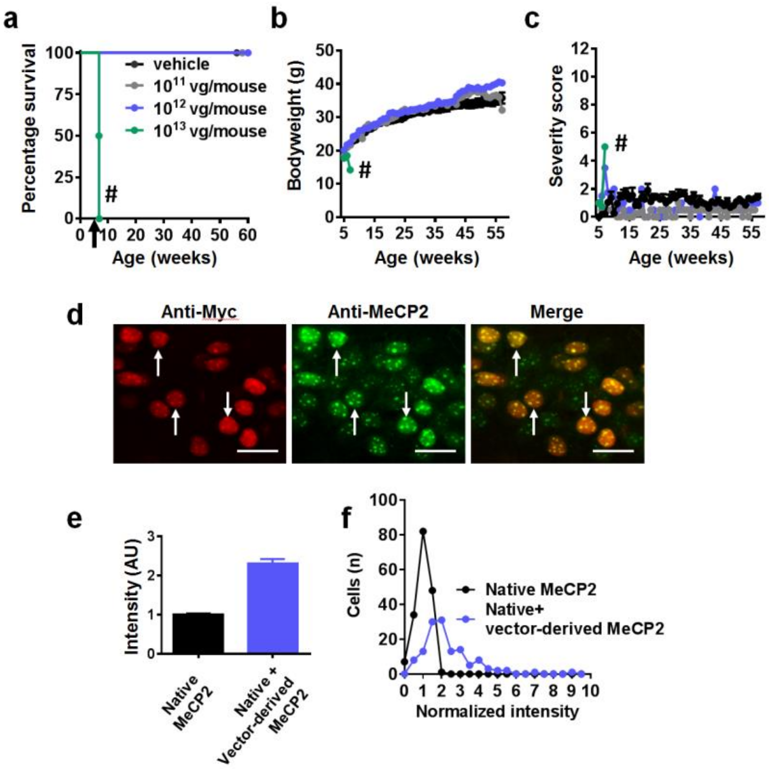

Figure S2. Systemic delivery of the 1st generation vector to wild-type mice is tolerated at low doses but toxic at high doses.

(a) Survival plot showing the early toxicity observed after IV injection of a $1x10^{13}$ vg/mouse dose of the 1st generation vector (green) compared to other doses and vehicle control. Arrow indicates age at injection. **(b-c)** Plots showing mean bodyweight and aggregate severity score, respectively, for these cohorts after injection. Data presented as mean ± SEM. **(d)** Flattened confocal stack images of the hippocampus CA1 region of wild-type mice injected with $1x10^{13}$ vg/mouse of the 1st generation vector. Tissues were immunolabelled with anti-Myc and anti-MeCP2 antibodies. White arrows indicate transduced cells. Scale bar indicates 20 µm. **(e)** Quantification of cellular levels of native MeCP2 and vector-derived MeCP2 in transduced and non-transduced cells in the hippocampus CA1 region of wild-type mice (n=2 mice; 131 transduced cells and 172 non-transduced cells). Data presented as mean ± SEM and normalised to native MeCP2. **(f)** Frequency distribution of normalised MeCP2 level in transduced and non-transduced cells. # indicates lethality at high dose.

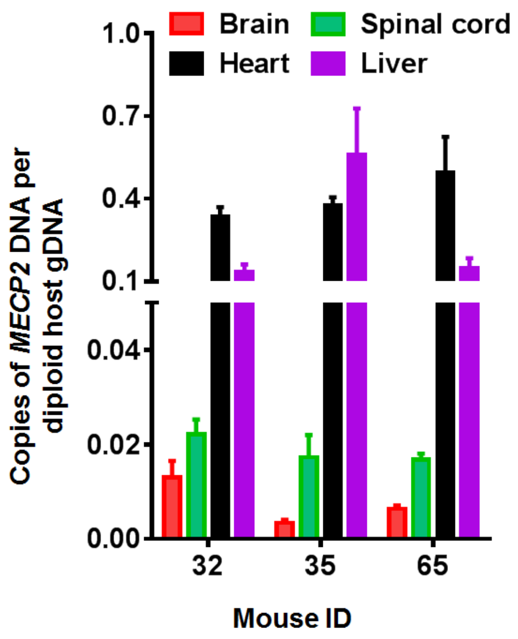

Figure S3. Biodistribution of 1st generation vector after intravenous injection.

Graph showing vector biodistribution in *Mecp2*$^{-/y}$ mice (n=3) as calculated by qPCR. Mice were injected intravenously at 5 weeks of age with 1x10^{12} vg/mouse and samples were taken approximately 22 weeks later. Data were standardised to host genomic DNA and are presented as mean ± SEM.

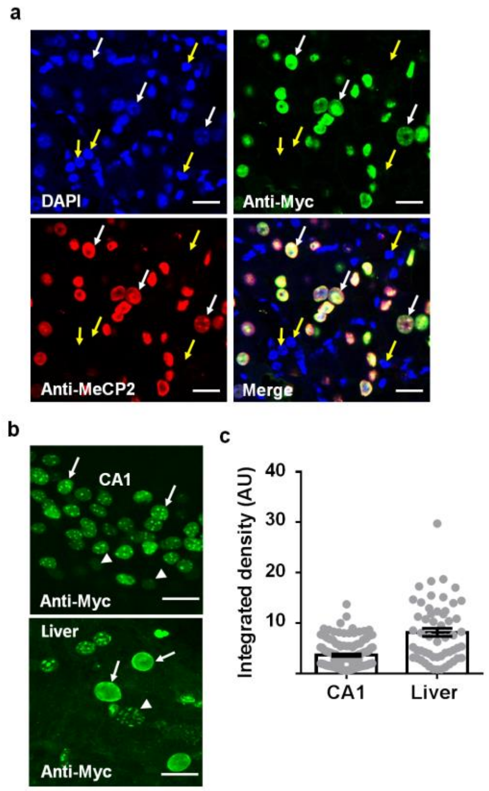

Figure S4. Intravenous injection of 1st generation vector resulted in high level of vector-derived MeCP2 expression in the liver.

(a) Representative confocal images of liver taken from WT mice injected intravenously with 1st generation vector at the dose of $1x10^{13}$ vg/mouse. Sections were immunolabelled with anti-Myc (green), anti-MeCP2 (red) and DAPI nuclear stain (blue). White arrows indicate transduced cells, whereas yellow arrows indicate non-transduced cells. **(b)** Flattened confocal stack images taken from the CA1 region of the hippocampus (top) and from the liver (mice were injected intravenously with $1x10^{13}$ vg/mouse) using the same confocal settings. Arrows indicate nuclei with a high level of vector-derived MeCP2 expression (based on fluorescence intensity of the anti-Myc antibody) and arrowheads indicate nuclei with low expression levels. Scale bar in (a) & (b) = 20 µm. **(c)** measurement of the integrated pixel intensity per nucleus in liver (55 transduced cells and CA1 (131 transduced cells) of the same mice (n = 3 mice). Data presented as mean ± SEM.

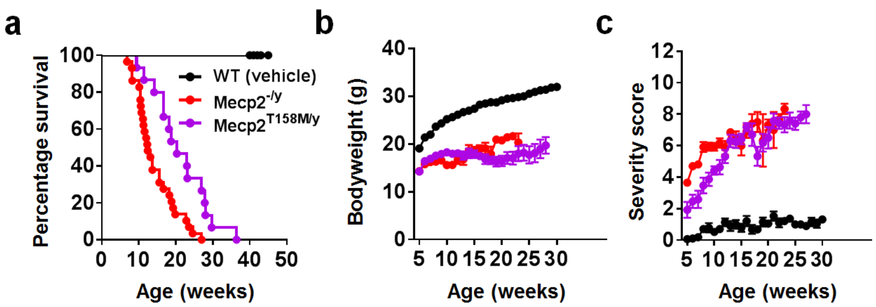

Figure S5. Comparison of *Mecp2$^{T158M/y}$* and *Mecp2$^{-/y}$* mice.

(a) Survival plot for *Mecp2$^{T158M/y}$* mice (n=15) and *Mecp2$^{-/y}$* mice (n=29). **(b-c)** Plots showing no significant differences in mean bodyweight and aggregate severity score, respectively, between *Mecp2$^{T158M/y}$* and *Mecp2$^{-/y}$* mice. Data presented as mean ± SEM.

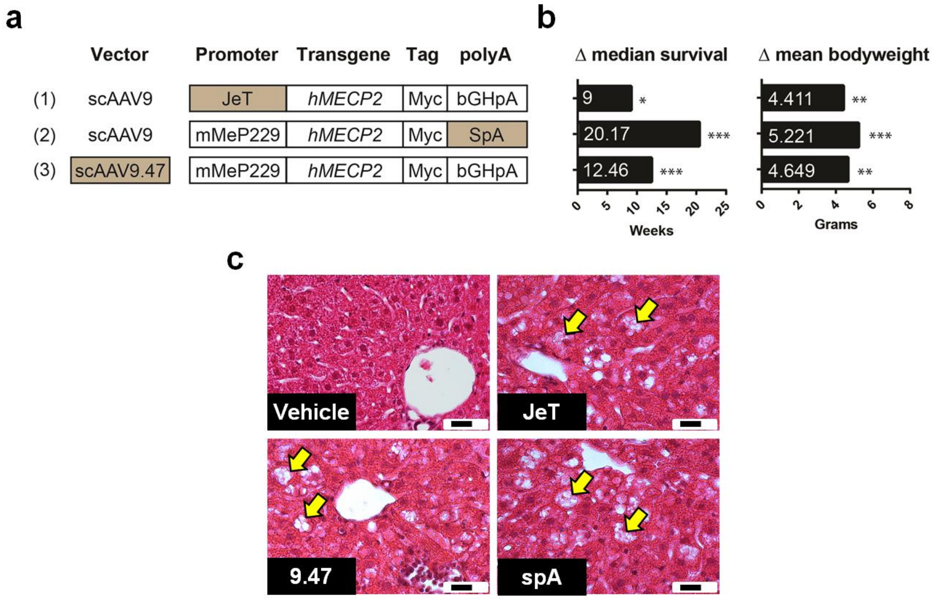

Figure S6. Novel vector design features, efficacy and liver phenotype.

(a) A summary of the design differences for three of the novel vectors described in the text. **(b)** Efficacy of these three novel vectors after intravenous injection of $1x10^{12}$ vg/mouse to 4-5 weeks old *Mecp2*$^{-/y}$ mice, , expressed as increase in median survival relative to the vehicle controls (left; compared using Mantel-Cox test) and mean bodyweight at the age of 11 weeks (right) relative to the vehicle controls (one-way ANOVA with Tukey's post-hoc pairwise comparisons). * $p < 0.05$, ** $p < 0.01$, *** $p < 0.001$. **(c)** Representative H&E-stained liver sections from mice injected with JeT, 9.47 or spA vectors. Arrows indicate vacuolation of hepatocytes; scale bar indicates 20 µm.

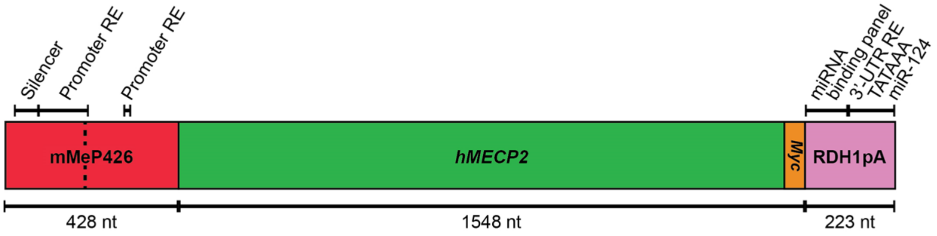

Regulatory element (RE)	Reference
Silencer	Liu & Francke (2006)*
Promoter RE	Adachi et al. (2005); Liu & Francke (2006)*; Liyanage et al. (2013)
miR-22 binding site	Feng et al. (2014)
miR-19 binding site	Jovicic et al. (2013)
miR-132 binding site	Klein et al. (2007)
3'-UTR RE	Coy et al. (1999); Newnham et al. (2010)*; Bagga & D'Antonio (2013)*
TATAAA polyadenylation signal	Coy et al. (1999); Newnham et al. (2010)*; Bagga & D'Antonio (2013)*
miR-124 binding site	Visvanathan et al. (2007); Jovicic et al. (2013)

Figure S7. Design of the 2nd generation vector construct.

Putative regulatory elements (RE) in the extended mMeP426 promoter and endogenous distal 3'-UTR are indicated. The extent of the mMeP229 promoter (used in the 1st generation vector) is indicated by the dashed line. Two non-endogenous cytosine nucleotides precede the ATG start codon. The RDH1pA 3'-UTR consists of several exogenous microRNA (miR) binding sites incorporated as a 'binding panel' adjacent to a portion of the distal endogenous *MECP2* polyadenylation signal and its accompanying regulatory elements. References with an asterisk indicate human *in vitro* studies, not rodent.
